# Protein Kinase C Regulates Meiosis in Mammalian Oocytes

**DOI:** 10.1002/bies.70087

**Published:** 2025-11-10

**Authors:** Jaroslav Kalous, Fatima J. Berro, Lucie Nemcova

**Affiliations:** ^1^ Laboratory of Biochemistry and Molecular Biology of Germ Cells Institute of Animal Physiology and Genetics Czech Academy of Sciences Rumburska 89, 27721 Libechov Czech Republic; ^2^ Laboratory of Developmental Biology Institute of Animal Physiology and Genetics Czech Academy of Sciences Rumburska 89, 27721 Libechov Czech Republic

**Keywords:** early embryo, meiosis, oocytes, protein kinase C, spindle

## Abstract

The protein kinase C (PKC) family comprises enzyme kinases that regulate cell survival, metabolism, and proliferation. PKC isotypes (PKCs) phosphorylate specific downstream substrates, thereby controlling critical steps in both mitotic and meiotic cell division. Throughout the cell cycle, PKCs orchestrate essential processes, such as chromosome segregation, recombination, and cell cycle progression. In vertebrates, PKCs play essential roles in oogenesis and the early stages of embryo development. Disruption of PKC signaling in mammalian oocytes can lead to errors in chromosome segregation and induce meiotic arrest. Therefore, investigating PKC function in meiosis is crucial for advancing fundamental biological research and for developing new approaches to infertility treatment.

## Graphical abstract

Protein kinase C (PKC) participates in the regulation of mammalian oocyte meiosis. PKC is involved in meiosis resumption in prophase I and in the stabilization of meiosis I and meiosis II spindles. PKC stimulates the maturation‐promoting factor during fertilization and stabilizes the mitotic spindle during early embryo development.

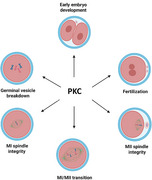

## 1 Introduction

Protein kinases C (PKCs) are phospholipid‐dependent serine/threonine kinases belonging to the AGC family of protein kinases [1]. PKCs, as crucial mediators in numerous signaling cascades, convert extracellular signals into intracellular responses [2]. PKCs regulate various cellular processes, such as proliferation, cell cycle progression, differentiation, apoptosis, and processes that may contribute to tumor formation and metastatic cancer cells dissemination [3, 4, 5]. In mammals, the protein kinase C (PKC) family comprises at least 12 isotypes, which are classified into three groups based on structural domains and activation mechanisms: conventional PKCs (cPKCs; α, β1, βII, γ), novel PKCs (nPKCs; δ, ε, η, θ, μ), and atypical PKCs (aPKCs; ζ, ι, λ) [6].

Like many kinases, PKCs are regulated by phosphorylation. In contrast to most kinases, these phosphorylations occur shortly after biosynthesis of PKCs [7, 8]. Newly synthesized PKCs are matured by phosphorylation at three conserved regions: the activation loop by the phosphoinositide‐dependent kinase PDK‐1 [9, 10] and two sites on the C‐tail region [8]. In most protein kinase C (PKC) isotypes, phosphorylation of the C‐tail sites relies on both the mammalian target of rapamycin complex 2 (mTORC2) [11] and the intrinsic catalytic activity of PKCs [12]. Phosphorylations of PKCs are constitutive and serve as a priming event that enables catalytic activation in response to cellular signaling [8, 13]

Phosphorylated PKC remains inactive and its activation depends on the intracellular second messengers such as diacylglycerol (DAG), calcium ions (Ca^2^⁺), and negatively charged phospholipids [14]. Specifically, cPKCs require both DAG and elevated intracellular Ca^2^⁺ levels in the presence of negatively charged acidic phospholipids [15]. Similarly, nPKCs also require both DAG and acidic phospholipids, however, they are thought to function independently of Ca^2^⁺ [16, 17]. However, aPKCs are activated only by acidic phospholipids, such as phosphatidylinositol(3,4,5)‐trisphosphate and phosphatidic acid, as well as by other lipids including ceramide and sphingosine‐1‐phosphate [18, 19, 20].

Upon activation, PKC isotypes are selectively translocated from cytoplasm to specific intracellular sites, including plasma membrane [21], nuclear membrane [22], endoplasmic reticulum [23], Golgi [24], mitochondria [25], and cytoskeletal components [26], where each isotype interacts with its anchoring protein, receptor for activated C kinase (RACK), and elicits different biological effects. Individual PKC isotypes target specific serine/threonine residues on specific protein substrates. Known PKC substrates include membrane‐associated myristoylated alanine‐rich C‐kinase substrate (MARCKS), receptor for activated C kinase (RACK), dynamin, the epidermal growth factor receptor (EGFR), mitogen‐activated protein kinase 5 (MEK5), cyclin‐dependent kinase 1 (CDK1), and cyclin B, among others [16, 27, 28, 29].

This review focuses on the role of PKCs in regulating oocyte meiosis and early embryo development.

## 2 PKC Regulates Cell Cycle Progression

In both mitosis and meiosis, the transition from the G2 phase to the M phase is driven by CDK1, the catalytic subunit of the M‐phase‐promoting factor (MPF) [30, 31, 32]. The MPF complex, which is composed of CDK1 and its regulatory subunit cyclin B, remains inactive until CDK1 is phosphorylated at Thr161 by a CDK‐inactivating kinase (CAK) and dephosphorylated at Thr14/Tyr15 by Cdc25 phosphatase [33, 34]. This phosphatase plays a key role in regulating cell division by removing inhibitory phosphorylation of CDK1, thereby activating it [35].

Other kinases that contribute to cell cycle regulation include Polo‐like kinase 1 (Plk1), mitogen‐activated protein kinase (MAPK), protein kinase B (PKB), Aurora kinases, CDK1‐activating kinases, and PKCs [36, 37, 38, 39, 40, 41, 42]. PKCs regulate multiple processes crucial for cell cycle progression, such as chromosome segregation, recombination, and differentiation, by phosphorylating specific target proteins [43]. The precise role of PKCs in cell cycle regulation varies across cell types and is context‐dependent. Depending on the timing of activation and the PKC isotypes involved, PKCs can either promote or inhibit the G1/S and G2/M transitions [4, 44]. They can suppress MPF activation by either inducing the CDK inhibitor p21 WAF1/CIP1, which blocks CDK1 activity, or by downregulating Cdc25 phosphatase, thereby preventing CDK1 dephosphorylation and activation [45]. On the other hand, PKCβ promotes progression through both the G1/S and G2/M transitions [46], and PKCδ stimulates the cell cycle of the mouse early embryo by phosphorylating and activating Cdc25B phosphatase [47].

## 3 Meiotic Arrest and Resumption of Meiosis

Meiosis is essential for both oogenesis and spermatogenesis. In mammalian oocytes, meiosis is initiated in the ovary during fetal development but is not completed until fertilization occurs in adulthood. Oocytes undergo two distinct meiotic arrests. The first meiotic arrest occurs in the ovary, where fully grown oocytes arrest at the diplotene stage of prophase I of the first meiotic division, allowing subsequent oocyte growth. The second arrest occurs at the metaphase II (MII) stage of the second meiotic division, when mature eggs await fertilization [48]. The first meiotic arrest is primarily maintained by high levels of cyclic adenosine monophosphate (cAMP), produced by the surrounding follicular cells. This keeps the oocyte dormant until hormonal signals trigger maturation [49, 50]. Upon release from prophase I, the fully grown oocyte must progress through two consecutive M phases without an intervening S phase.

In the preovulatory follicle, meiotic re‐entry is marked by germinal vesicle breakdown (GVBD), followed by microtubule assembly around the chromosomes and the formation of a bipolar spindle at metaphase I (MI) [51]. After extrusion of the first polar body and completion of meiosis, the oocyte arrests again at MII. Meiosis II resolves only upon gamete fusion at fertilization [52, 53]. Release from the first meiotic arrest and resumption of meiosis require the activation of MPF [30, 54, 55]. The Cdc25B phosphatase plays a key role in regulating the re‑initiation of meiosis by removing the inhibitory effect of CDK1 [56]. Alterations in CDK1 activity and/or cyclin B1 levels have the potential to disrupt meiotic progression [57, 58, 59].

## 4 PKC Affects Intercellular Communication in Cumulus‐Enclosed Ooocytes

In the mammalian ovary, the oocytes and the surrounding cumulus and granulosa cells are interdependent, playing essential roles in follicular development and oocyte ovulation. Within the follicle, cumulus and granulosa cells communicate closely via intercellular channels called gap junctions, which allow the passage of nutrients, ions, and inhibitory signals from somatic cells to the oocytes. This exchange supports the metabolic needs of growing mouse oocytes and maintains meiotic arrest [60, 61]. Studies in rat oocytes have shown that gap junction communication is modulated by various factors that regulate the expression and phosphorylation status of connexin 43 (Cx43), a key protein involved in gap junction structure [62].

Phosphorylation of Cx43 is associated with the closure of gap junctions, which may be sufficient to trigger oocyte meiotic resumption by blocking the passage of inhibitory signals to the oocyte [63]. Several kinases, including protein kinase A (PKA), PKC, glycogen synthase kinase 3 (GSK3), and MAPK, have been shown to phosphorylate Cx43 in mice and rat oocytes [64, 65].

In vivo, LH‐induced phosphorylation of Cx43 in cumulus cells results in decreased levels of cAMP and cyclic guanosine monophosphate (cGMP) in oocytes. This decline initiates GVBD and meiotic resumption in mouse oocytes [63, 64, 66]. PKC signaling has been identified as the main pathway mediating the betacellulin‐induced phosphorylation of Cx43 at Ser368 in human granulosa cells [67]. In FSH‐stimulated mouse cumulus‐enclosed oocytes, the PKC epsilon (PKCε) isotype phosphorylates Cx43, thereby ceasing the transfer of key molecules through gap junctions before meiosis resumption [68].

## 5 PKC Participates in Meiosis Regulation

In mouse oocytes, PKC isotypes influence nearly every stage of meiosis by participating in signaling pathways that regulate meiotic resumption and progression, oocyte maturation, chromosome dynamics, formation of a bipolar spindle at MI and MII, completion of meiosis II, oocyte activation at fertilization and initiation of the first mitotic division [69, 70, 71, 72, 73]. In mouse GV oocytes, pPKC localizes throughout the entire GV and after GVBD, pPKC is distributed around chromosomes. During MI and MII, pPKC localizes on the entire meiotic spindle [74].

The spatiotemporal distribution of individual PKC isotypes plays a key role in regulating meiotic resumption. Upon activation, PKC isotypes exhibit distinct localization patterns supporting the idea that each isotype plays a specific role at different stages of oocyte development [69, 75].

The expression of conventional PKCs (α, β, γ), novel PKCs (δ, ε, μ), and atypical PKCs (λ, ζ) has been detected in rat eggs [76]. In mice, PKCα, γ, δ, ζ, and ε isotypes are present in the cumulus cells surrounding oocytes, while PKCα, γ, δ, and ζ have been confirmed in zona pellucida‐free oocytes [77]. These findings suggest that PKC isotypes may play different roles depending on whether oocytes are enclosed by cumulus cells or not. Specifically, activated PKC is essential for resuming meiosis in cumulus‐enclosed mouse oocytes. The effect of PKC activators and inhibitors on oocyte maturation in vitro depends on the presence of cumulus cells [78]. Activation of PKC in bovine cumulus‐enclosed oocytes has been shown to accelerate GVBD [79]. In contrast, in cumulus‐free mouse oocytes, active PKC inhibits the resumption of meiosis [75, 77, 80]. Although depletion of PKCβI does not impair resumption of meiosis in mouse oocytes, the PKCβI overexpression delays GVBD by modulating CDK1 activity and cyclin B1 levels [69]. In mouse oocytes, just before GVBD, all conventional PKC isotypes, except PKCγ, relocate from the cytoplasm to the nucleus, and this translocation is essential for GVBD induction [71, 75, 80, 81]. Targeted antibody‐mediated inhibition of PKCα and PKCβII within the nucleus significantly reduces the GVBD rate in mouse oocytes [71]. As PKCβI colocalizes with lamin A/C on the nuclear envelope of mouse oocytes, it can be assumed that the translocated PKCs participate in the phosphorylation of nuclear membrane lamins, which are key structural components of the nuclear envelope in oocytes [71]. PKCβI‐mediated lamin phosphorylation may contribute to germinal vesicle disassembly during meiosis reinitiation in mice [81] (see Table 1).

**TABLE 1:** Role of PKC in meiosis.

Role of PKC	Reference
PKCβI regulates Cyclin B1 level and CDK1 activity during G2/M transition	[69]
PKCβI is involved in the nuclear envelope disintegration	[81]
PKCδ is essential for MI‐to‐MII transition	[70]
PKCβI is required for MI and MII spindle organization	[69]
PKCδ and PKCζ stabilize MII spindle	[73, 82]

It has been suggested that PKC plays distinct roles in oocytes and the surrounding follicular somatic compartment by regulating MAPK phosphorylation. In the absence of cumulus cells, PKC activation in rat and mouse cumulus‐free oocytes induces downregulation of MAPK phosphorylation and inhibits resumption of meiosis [83, 84]. By contrast, when cumulus cells are present, PKC activation within the cumulus cells of mouse cumulus‐enclosed oocytes enhances MAPK phosphorylation and induces GVBD [29].

During gonadotropin‐induced meiotic maturation in porcine and mouse oocytes, EGFR activity in the ovarian follicle, specifically in mural granulosa and cumulus cells, is essential for oocyte maturation, cumulus expansion, and ovulation [85, 86]. In porcine granulosa cells, EGFR signaling activates the MAPK pathway, promoting the retraction of transzonal projections during meiotic resumption and enhancing fertilization potential [87]. In follicle‐stimulating hormone (FSH)‐treated porcine cumulus‐enclosed oocytes, PKC signaling activates EGFR, contributing to FSH‐induced meiotic resumption [88]. In porcine cumulus cells, MAPK activation occurs through PKC‐dependent EGFR transactivation [89]. In mouse cumulus‐enclosed oocytes, inhibition of two novel isotypes, PKCδ and PKCθ, blocks FSH‐induced oocyte maturation in vitro. This blockade can be partially reversed by amphiregulin, confirming the involvement of PKCδ and PKCθ in the EGF signaling axis [90].

## 6 PKC Contributes to Meiotic Spindle Integrity

**FIGURE 1:**
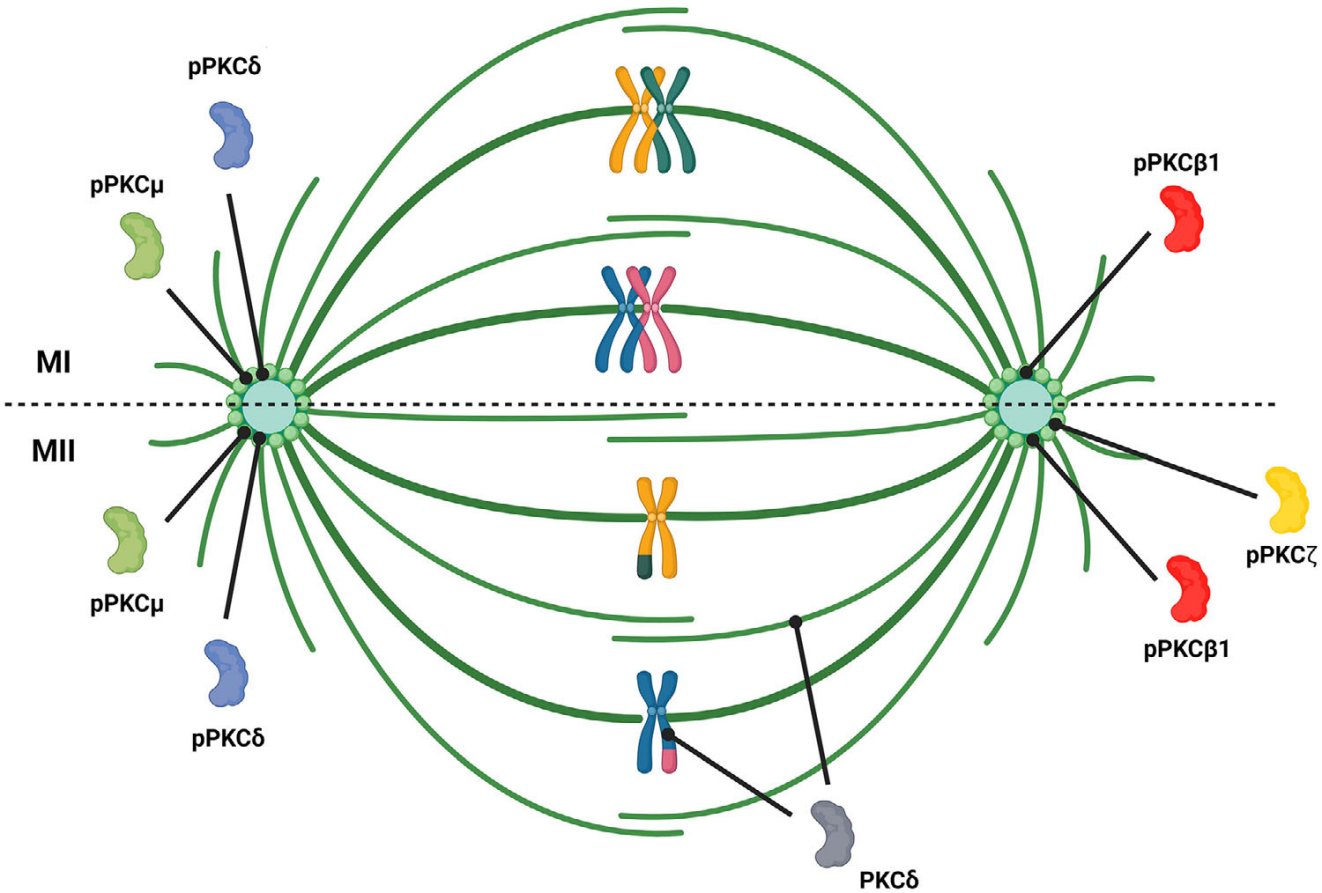
Localization of PKC isotypes on the meiotic spindle. Phosphorylated PKCμ, PKCβ, and PKCδ are localized at the spindle poles during meiotic metaphase I and metaphase II. Phosphorylated PKCζ is concentrated specifically on the metaphase II spindle poles. PKCδ is also found on metaphase II chromosomes and spindle microtubules. PKC, protein kinase C.

PKC isotypes play a crucial role in meiosis by participating in signaling pathways that regulate its progression. Studies on mouse oocytes revealed that these factors contribute to oocyte maturation, chromosome dynamics, and the formation of a bipolar spindle during meiosis I and II. PKCs also play a role in the completion of meiosis II and the initiation of the first mitotic division following mouse oocyte activation at fertilization [69, 70, 91, 92]. PKC activity is tightly regulated to ensure the proper timing of meiotic transitions. Dysregulated PKC signaling in mouse oocytes can lead to errors in chromosome segregation and induce meiotic arrest [43, 69]. During MI and MII, phosphorylated PKC isotypes predominantly localize at the spindle poles (see Figure 1).

Following GVBD, PKCβI is evenly distributed in the cytoplasm and becomes phosphorylated in metaphase. Phosphorylated PKCβI (pPKCβI) then accumulates at spindle poles and subsequently localizes to the midbody during telophase in mouse oocytes [69]. Depleting PKCβI in mouse oocytes using siRNA results in defective spindle formation, spindle checkpoint activation, MI arrest, and failure of first polar body extrusion. Additionally, PKCβI knockdown also results in abnormal spindle morphology, chromosome misalignment, and meiotic arrest at MI [69]. PKCα localizes to the meiotic spindle of mouse oocytes during MII [93], and PKCδ binds to the spindle during the MI‐to‐MII transition, aligning with chromosomes at MII [94].

During both the MI‐ and MII‐stages, phosphorylated PKCδ (pPKCδ) accumulates at the spindle poles of mouse oocytes and co‐localizes with pericentrin and γ‐tubulin in the MTOCs, thereby supporting spindle organization [72]. The loss of regulatory control over PKCδ impairs the MI‐to‐MII transition, resulting in the premature meiotic exit in mouse oocytes [70].

In mammalian oocytes, which form acentrosomal spindles, phosphorylated PKCζ (pPKCζ) assembles into a ring at each spindle pole. This suggests that PKCζ may play a role in stabilizing the barrel‐shaped spindle in the absence of centrioles of mouse oocytes [82]. In fertilization‐competent eggs, arrested at MII, pPKCζ remains associated with the spindle until telophase II. When pPKCζ is inhibited, spindle microtubules are disrupted and chromosome misalignment occurs. These findings highlight the essential role of PKCζ in spindle stability in mouse eggs [73]. PKCζ may regulate spindle stability by phosphorylating MTOC components and/or spindle‐associated MAP kinases in mouse oocytes [73].

Knockdown of protein kinase D1 (PKD1; formerly PKCμ) does not interfere with meiotic resumption in mouse oocytes, however, PKD1 contributes to oocyte maturation by regulating both spindle organization and actin filament distribution [95]. Phosphorylated PKD1 localizes to spindle poles and may influence cytoskeletal dynamics, as a loss of PKD1 function induces cytoskeletal defects during meiosis [95].

## 7 Importance of PKC in Fertilization‐Competent Eggs

**I**t has been suggested that fertilization‐competent eggs, arrested at the meiotic MII stage exhibit distinct localization patterns of individual PKC isotypes. Immunostaining with a PKC antibody, capable of detecting both phosphorylated and non‐phosphorylated PKC isotypes, revealed the presence of PKCα, PKCγ, PKCδ, and PKCζ in the cytoplasm and on the MII spindle of fertilization‐competent eggs [73].

FRET analysis suggests that PKCζ and PKCδ may interact with α‐tubulin within the meiotic spindle of mouse eggs. Furthermore, PKCζ and PKCδ are in close molecular proximity to α‐tubulin, suggesting a potential functional interaction [73, 82]. These findings indicate a possible involvement of PKCζ and PKCδ in the stability and organization of the meiotic spindle. In mouse eggs, phosphorylated PKCζ (p‐PKCζ) accumulates at the ends of the acentrosomal spindle, while total PKCζ is distributed along the entire spindle apparatus [96]. The inhibition of p‐PKCζ appears to rapidly disrupt the meiotic spindle, highlighting its specific role in spindle stability. However, inhibition of other active PKC isotypes in mouse eggs does not result in spindle disassembly, suggesting that these isotypes may contribute to distinct aspects of the egg‐to‐zygote transition [82, 97]. Furthermore, p‐PKCδ colocalizes with p‐PKCζ at the spindle poles, indicating a potential role for p‐PKCδ in stabilizing MII‐spindle in mouse eggs [72]. It has been proposed that siRNA‐mediated silencing of PKCδ disrupts the spindle apparatus in mouse eggs, thereby confirming the role of PKCδ in spindle positioning and organization [72]. However, it is important to note that PKCδ knockout mice exhibit no apparent decline in viability and fertility, suggesting that compensatory pathways may be involved in maintaining MII‐spindle integrity [98]. In summary, the presented findings identify PKCζ and PKCδ as significant regulators of MII spindle stability, while other PKC isotypes appear to have distinct functions during fertilization and the egg‐to‐zygote transition.

## 8 Role of PKC in Fertilization

Fertilization induces a series of structural and biochemical changes within the egg as a single sperm cell penetrates and initiates zygote formation. This critical developmental step relies on a series of signal transduction events initiated by sperm penetration. As illustrated in Table 2, PKC signaling plays a key role in regulating critical egg activation processes during mammalian fertilization (see Table 2).

**TABLE 2:** PKC regulates fertilization of MII eggs.

Role of PKC	Reference
Activation of PKC is essential for the transition of the mouse egg into the zygote	[99]
PKC regulates distribution of endoplasmic reticulum in bovine activated eggs	[100]
PKCβ regulates nuclear function and cortical granules exocytosis in porcine MII eggs	[101]
In fertilized mouse oocytes, PKCα and PKCβ1 are involved in oocyte activation	[75, 93]
During fertilization of mouse oocytes, PKCδ phosphorylates the Cdc25B Ser96 residue	[27, 102]
Depletion of PKCδ disrupts fertilization and early embryonic development in mice	[94]
PKCδ modulates MPF activity in one‐cell stage mouse embryos	[103]

During fertilization, the exocytosis of cortical granules is a process primarily mediated by PKC, essential for ensuring monospermic fertilization and proper early embryonic development. In unfertilized eggs, cortical granules are associated with serine proteases. Upon fertilization, cortical granules release their contents into the perivitelline space through a calcium‐dependent process known as the cortical reaction. This reaction modifies the oocyte's zona pellucida, the glycoprotein envelope, thereby blocking further sperm binding and penetration, and thus ensuring monospermic fertilization and proper development of the early embryo [104].

**FIGURE 2:**
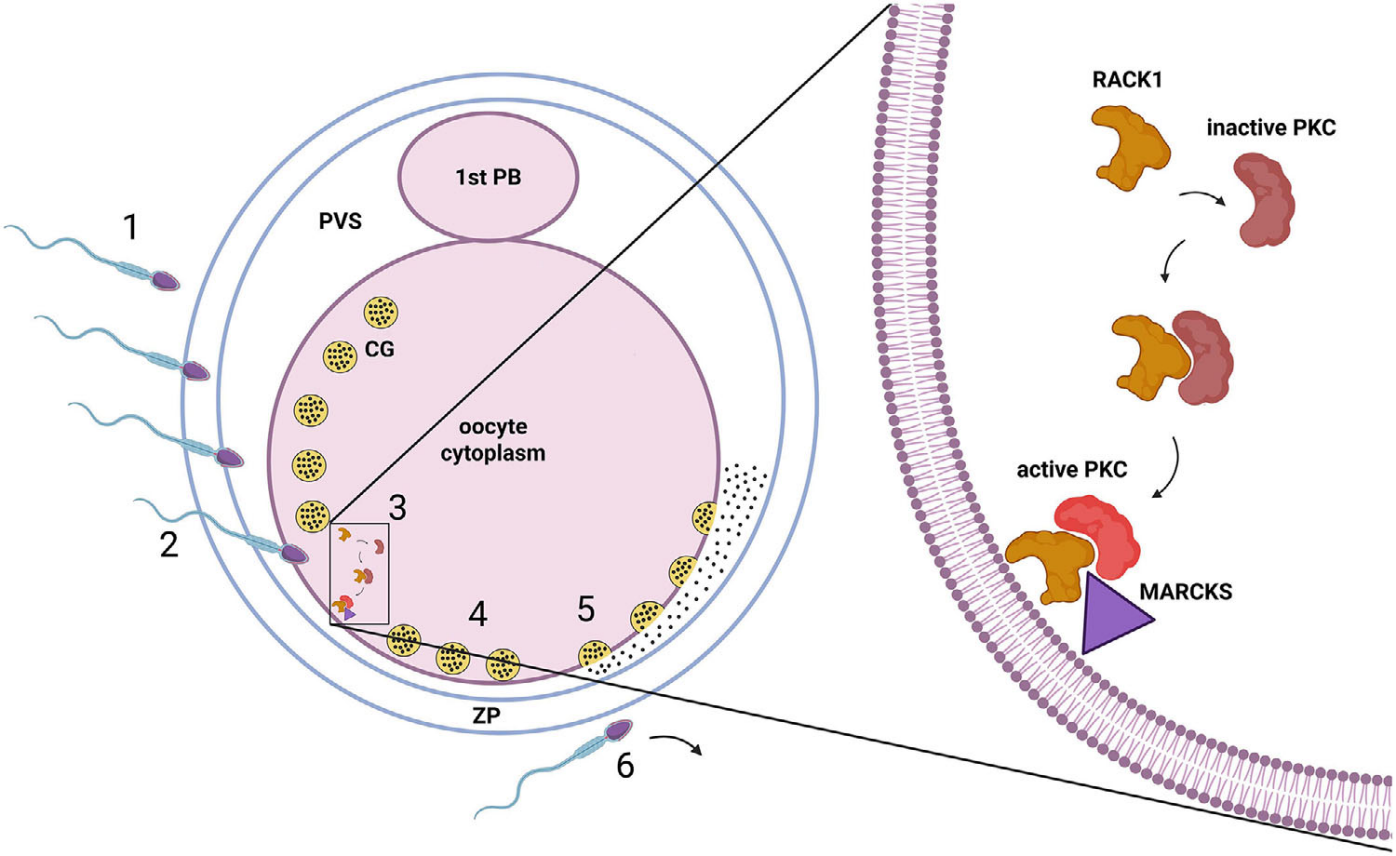
The role of PKC in inducing cortical reaction. (1) The sperm moves toward the egg. (2) The sperm penetrates the zona pellucida (ZP) and the plasma membranes of the sperm and egg fuse, activating the egg. (3) Upon egg activation, the receptor for activated C kinase 1 (RACK1) recruits protein kinase C (PKC) in the cytoplasm. This complex then moves to the plasma membrane, where PKC interacts with and phosphorylates myristoylated alanine‐rich C‐kinase substrate (MARCKS). (4) Active MARCKS induces changes in the actin cytoskeleton, enabling cortical granules (CG) to reach the plasma membrane. (5) CG fuse with the plasma membrane and the CG content is released into the perivitelline space (PVS) by exocytosis in a process known as the cortical reaction. (6) The cortical reaction induces biochemical changes in the ZP to establish a polyspermy block.

In mouse eggs, fertilization induces the translocation of conventional PKC isotypes (PKCα, β, and γ) to the plasma membrane, positioning them optimally for participation in cortical granule exocytosis [73, 76, 101]. As documented in rat eggs, translocation of PKC to the egg cortex is enabled by RACK1, a scaffolding protein that binds cytoplasmic PKC upon egg activation. Then RACK1 shuttles the PKC to the egg cortex, and PKC is recruited to the egg plasma membrane to be activated. Subsequently, PKC is ready to phosphorylate MARCKS proteins, which interact with the actin cytoskeleton and can crosslink actin filaments into a network at the cell cortex [105, 106]. Following the phosphorylation of MARCKS by PKC, the actin network may undergo disassembly [107]. Disassembly of the actin network in the cortex has been shown to result in the release of cortical granules from the cortical actin network, thereby enabling them to reach the plasma membrane for exocytosis [101, 107, 108] (see Figure 2).

After fertilization of mouse eggs, PKCα, β, and γ isotypes are translocated from the cytosol to the plasma membrane. At this point, these conventional PKCs may interact with DAG and phospholipids to become activated [91, 109]. Similarly, in PMA‐activated MII human eggs, conventional PKCα, β1, and γ isotypes are translocated to the plasma membrane [110]. These data suggest that conventional PKC isotypes, which initiate the cortical reaction in the egg cortex at fertilization, persist on the egg membrane after fertilization and contribute to the egg‐to‐zygote transition.

## 9 PKC Regulates Early Embryo Development

The initial phase of embryo development is started by the fertilization of MII‐stage oocytes. This phase involves critical processes such as zygote division, the maternal‐to‐zygotic transition, blastocyst formation, and cell fate determination. Representatives of all three categories of PKCs—conventional: PKCα and PKCγ, atypical: PKCζ and PKCλ, and novel: PKCμ and PKCδ—are present in unfertilized mouse eggs and in the developing mouse embryos up to the eight‐cell stage [80, 111, 112].

After fertilization, different PKC isotypes have been observed to localize in various regions, such as the cortex, spindle, polar body contractile ring, and female pronucleus [75, 82, 113]. The distinct localization of various PKC isotypes during the post‐fertilization development of the zygote and preimplantation embryo suggests that they may play different roles in converting the fertilized egg into a zygote. Due to the diverse functions of the different PKC isotypes, it is predicted that some isotypes will exhibit distinct subcellular localizations at critical stages of early development [82, 99] (see Table 3).

**TABLE 3:** Role of PKC during early embryo development.

Role of PKC	Reference
PKC activation induces pronucleus formation in rat fertilized eggs	[116]
PKCδ supports the first cell cycle of mouse embryos by phosphorylating Cdc25B phosphatase	[102]
Inhibition of PKCδ prevents development of bovine embryos	[117]
PKCδ promotes mouse blastocyst implantation	[94, 118]
PKC signaling regulates tight junction membrane assembly in the pre‐implantation mouse embryo	[119]
Ezrin phosphorylated by PKC promotes embryonic attachment in mice	[120]
PKCι isotype phosphorylates ezrin, which is important in eight‐cell mouse embryo compaction	[121]

In fertilized mouse eggs, both PKCζ and p‐PKCζ remain associated with the spindle from MII to anaphase II, and they associate with midzone microtubules during anaphase II and telophase II, suggesting that PKCζ plays a role in spindle stability [73]. Following the fertilization of mouse and rat eggs, PKC plays an important role in the initial stages of egg activation, and PKC activity is essential for transforming the egg into a zygote [114, 115]. Pronucleus formation in fertilized rat eggs may be promoted by PKC activation and MAPK dephosphorylation [116]. However, prolonged PKC activation using PMA has been shown to block cleavage in the early mouse embryo [80]. The regulatory role of PKC family members in early embryo development has been the subject of detailed studies (see Table 3).

In mouse eggs, following fertilization, the conventional PKCα, β, and γ isotypes translocate from the cytosol to the plasma membrane. In this environment, they can interact with DAG and phospholipids, undergo activation, and thereby contribute to egg activation [73, 91, 109]. Similarly, PKCα, β1, and γ isotypes are translocated to the plasma membrane in PMA‐activated MII human eggs [110].

The role of the novel PKCδ isotype in egg fertilization and zygote development has been thoroughly studied (see Table 3). PKCδ activates MPF at fertilization, and MPF activity is essential for the transition from the one‐cell stage to the two‐cell stage [103]. After fertilization, PKCδ translocates to the spindle midzone, suggesting its involvement in the transition from meiosis to mitosis. PKCδ is then rephosphorylated during the M‐phase of the first mitotic cleavage division [122]. After the first mitosis, PKCδ becomes dephosphorylated and accumulates in the pronuclei and nuclei of 2‐cell‐stage mouse embryos, together with PKCγ, α, and ζ [113]. During the early stages of embryonic development, PKCδ plays a crucial role in maintaining the integrity and proper function of the spindle apparatus [91]. In cattle, PKCδ is essential for the development of preimplantation embryos, and inhibition of PKCδ prevents bovine embryos from progressing beyond the 8‐ to 16‐cell stage [117]. Additionally, inhibiting PKCδ at or after the 16‐cell stage reduces blastocyst development rates, total blastomere numbers, and the inner cell mass‐to‐trophoblast cell ratio [117]. PKCδ knockout in mice leads to impaired fetal development and is associated with hyperplasia of heart elastic fibers and lung inflammation [118].

Active p‐PKCζ is detected in both pronuclei 6 h after fertilization, remains enriched in both pronuclei at 14 h post‐fertilization, and is also present in the nuclei of the two‐cell embryo [73]. After the 2‐cell stage, a significant decrease in the nuclear localization of PKCα, PKCγ, and PKCδ is observed, whereas PKCζ remains localized along the nuclear periphery [111]. Inhibiting PKCζ, but not other PKC isotypes, causes a rapid disruption of the meiotic spindle. This finding suggests that PKCζ is involved in spindle stability, while other PKC isotypes may have distinct roles in the egg‐to‐zygote transition. During the late eight‐cell stage, just before compaction, the PKCζ isotype becomes highly concentrated in the nuclei [111]. However, during embryo compaction, the PKC isotypes α, γ, δ, and μ delineate the cell boundaries to varying degrees and are largely absent from the nucleus [111, 123].

In experimental settings, the activation of PKC isotypes by the natural agonist leads to the migration of PKCα to the internal cell‐cell boundaries. PKCβI has been observed to accumulate specifically in the nuclei of 4‐cell‐stage embryos. During the post‐compaction stage, PKCβII is consistently distributed throughout the cytoplasm and nuclei, persisting from the 4‐cell stage to the blastocyst stage [112]. It has been proposed that blocking both PKCδ and PKCε with peptides that interfere with adapter sites disrupts the movement of these isotypes, potentially leading to altered transcription [112]. This suggests that the specific localization of a PKC isotype within the cells may influence its activity, and isotypes localized in the nucleus are likely involved in transcription during early embryo development.

In the late 8‐cell stage mouse embryo, the atypical PKCζ and PKCλ isotypes phosphorylate ezrin, a protein involved in cell signaling and structure. Ezrin is often associated with cell membranes and the cytoskeleton, and its function is important for cell polarization and embryo compaction [121]. Additionally, ezrin expression is maintained during the later stages of embryo development, when cells differentiate into the trophectoderm and trophoblast. It has been suggested that, when phosphorylated by PKC, ezrin may promote embryonic attachment by regulating the invasiveness of trophoblast cells, a process essential for embryo implantation and placental development [120, 124]. In the pre‐implantation mouse embryo, PKC signaling plays a role in regulating tight junctions assembly. These findings suggest that at least two atypical PKC isotypes, PKCλ and PKCζ, may contribute to the control of tight junctions assembly in the trophectoderm [119].

## 10 Conclusion

PKC has been identified as a critical regulator of both the mitotic and meiotic cell cycles. Individual PKC isotypes exert their effects by interacting with multiple targets, including MPF, lamins, and microtubules. This review highlights the role of PKC in regulating oocyte meiosis and early embryo development. PKC isotypes have been shown to support meiosis reinitiation, promote meiotic progression, and play a regulatory role in early embryonic development. Dysregulation of PKC activity during oocyte meiosis can result in defective spindle assembly, errors in chromosome segregation, and compromised preimplantation development, ultimately leading to infertility or birth defects. Identifying specific substrates of individual PKC isotypes could provide new opportunities to control meiotic progression and early development. A more profound understanding of PKC's multifaceted functions and manifold roles of PKC in oocyte meiosis and early embryo development is imperative for advancing reproductive biology and developing novel infertility therapies. In the context of age‐related fertility decline and potential therapeutic applications, future research could explore methods to maintain or restore PKC levels in oocytes to improve fertility outcomes.

## Author Contributions

J.K.: Writing, original draft preparation, and editing. L.N.: Writing and editing. F.J.B.: Editing and figure design.

## Conflicts of Interest

The authors declare no conflicts of interest.

## Data Availability

Data sharing not applicable to this article as no datasets were generated or analyzed during the current study.
